# Editorial: Abiotic stress: Molecular genetics and genomics, volume II

**DOI:** 10.3389/fpls.2022.1101139

**Published:** 2023-01-18

**Authors:** Rohini Garg, Prasanta K. Subudhi, Rajeev K. Varshney, Mukesh Jain

**Affiliations:** ^1^ Department of Life Sciences, School of Natural Sciences, Shiv Nadar University, Gautam Buddh Nagar, India; ^2^ School of Plant, Environmental, and Soil Sciences, Louisiana State University Agricultural Center, Baton Rouge, LA, United States; ^3^ Centre for Crop & Food Innovation, State Agricultural Biotechnology Centre, Food Futures Institute, Murdoch University, Perth, WA, Australia; ^4^ School of Computational & Integrative Sciences, Jawaharlal Nehru University, New Delhi, India

**Keywords:** abiotic stress, functional genomics, gene regulatory networks, climate change, comparative transcriptomics, genome-wide association studies, phylogenetic analysis

Abiotic stresses constitute major threat to farming system worldwide. The ongoing climate change is further exacerbating the global farming landscape due to increased frequency and intensity of abiotic stresses leading to reduced productivity and stability in crop plants (Ashikari and Ma, 2015; Hussain et al., 2019). In recent years, there has been a remarkable improvement in crop productivity due to development and implementation of innovative breeding and genetic tools and technologies (Varshney et al., 2021). However, increasing human population and rising living standards are expected to increase the global food demand in coming years. Therefore, further agricultural innovations are required to meet this challenge. Since most cultivars have been developed to perform well under optimal environments with minimal perturbations (Kukal and Irmak, 2018; Bharadwaj et al., 2021), there is a need for more stress-tolerant crop varieties to sustain crop productivity under adverse environments (Raza et al., 2021).

Climate variations threaten both global food security (Godfray et al., 2010) and sustainability of the farming system (Porfirio et al., 2018). The transformation of agriculture towards sustainability inspires research to mitigate the impact of climate change induced abiotic stresses (Wheeler and Von Braun, 2013). Among others, use of cultivars with enhanced adaptation to abiotic stresses is the most logical and economical approach to have significant impact on both sustainability and food security at a global scale (Chaturvedi et al., 2017). Therefore, understanding the plants’ response to various abiotic stresses at the whole genome level, discovery and characterization of important natural variants, and elucidation of abiotic stress adaptation mechanisms using modern genomic tools is crucial for designing next-generation climate-resilient crop cultivars (Garg et al., 2016; Palit et al., 2020; Rajkumar et al., 2022).

The current Research Topic “Abiotic Stress: Molecular Genetics and Genomics, Volume II” encompasses a collection of 30 original research articles and one review on wide range of topics, such as genome-wide survey of key abiotic stress tolerance genes and their characterization, genome-wide association studies (GWAS), comparative genome-scale transcriptomic, ionic, degradome, microRNA profiling, selection of key salt tolerance genes during breeding process, and abiotic stress tolerance mechanisms. These research articles provide novel insights into plants’ responses and adaptation to several abiotic stresses, such as salinity, alkalinity, drought, temperature extremes, nutrient and metal toxicity, in a variety of plant species.

## Gene family analysis for discovery of candidate gene(s) implicated in abiotic stress tolerance

The analysis of a set of evolutionary related homologous genes (gene families) can provide important insights into their distinct/overlapping functions and identify candidate gene(s) involved in important biological processes including abiotic stress response (Jain et al., 2010; Singh and Jain; Singh et al., 2017). The article by Wang et al. reported identification of 24 metal tolerance protein (MTP) encoding genes in peanut (*AhMTP*) in a genome-wide survey followed by detailed phylogenetic relationship, gene structure, protein structure, and gene expression analyses. The authors reported that the differential response of *AhMTP* genes to Fe, Cd, and Zn exposure in two peanut cultivars with contrasting response to metal toxicity may be due to differential metal translocation from roots to shoots. In another study, Yuan et al. identified 39 members of cysteine synthase (*CSase*) gene family in alfalfa (*Medicago sativa* L.). The authors performed a systematic phylogeny, gene structure, conserved domain and synteny analysis of this gene family. The overexpression of a *CSase* gene improved alkali tolerance by increasing the antioxidant and osmolyte production in alfalfa. A genome-wide analysis of late embryogenesis abundant (LEA) proteins in mung bean, adzuki bean and cowpea by Singh et al. provided insights into their structural and functional diversity in the three *Vigna* species. One interesting finding of this study was that LEA-6 group was missing in the mung genome and all seven groups were preset in the cowpea genome. The gene expression studies involving seven mung bean genes demonstrated their role in heat stress response. In a comprehensive survey of soybean genome, Xu et al. identified 22 TLP (tubby-like protein) genes which were analyzed for their phylogenetic relationship, gene structure and motif analyses. The functional characterization of a candidate gene, *GmTLP8*, demonstrated its role in drought and salinity stress responses by triggering the downstream stress-responsive genes. Likewise, another genome-wide study by Liu et al. identified 38 *GmTIFY* transcription factor genes. The overexpression of two of these genes (*GmTIFY10e* and *GmTIFY10g*) in transgenic *Arabidopsis* and soybean plants showed improved salt tolerance compared with wild-type plants, whereas the RNAi lines exhibited enhanced sensitivity to salt stress. Further, evidence of the involvement of these genes in the ABA signaling pathway was also provided.

The study by Huang et al. reported identification of 26 members of the plant-specific Teosinte Branched1/Cycloidea/Proliferating Cell Factor (TCP) transcription factor family in Pak-choi [*Brassica campestris* (syn. *Brassica rapa*) ssp. *chinensis* var. communis]. The gene expression analysis revealed the differential expression of several members in response to different types of abiotic stresses. In another article, Li et al. provided a comprehensive analysis of cysteine-rich polycomb-like protein (CPP) family transcription factors in *Brassica napus* and its two diploid progenitors. The analysis suggested that whole genome duplication and transposed duplication might be responsible for the expansion of CPP gene family in *B. napus* during allopolyploidization and few of *BnCPP* genes undergo neo- or sub-functionalization. The expression analysis under salinity stress revealed the adaptive advantage of allopolyploid *B. napus* compared with the diploid progenitors.

The study by Yu et al. reported 37 Calcineurin B-like protein-interacting protein kinase (CIPK) encoding genes in the ornamental woody plant *Lagerstroemia indica* and performed various analyses thereof. The collinearity and synonymous substitution rate analyses revealed that most of duplicated *LiCIPK*s were retained by the two whole genome duplication events. Several *LiCIPK*s exhibited differential expression under different abiotic stress conditions. Further, role of *LiCIPK30* in improving salt and osmotic tolerance was demonstrated *via* its overexpression in *Arabidopsis*. The above studies provide an important resource for the prioritization of candidate genes for further investigations into their function and mechanism of action in abiotic stress responses.

## Candidate gene based studies for improvement of abiotic stress tolerance

Chang et al. successfully cloned and characterized a *WUSCHEL-RELATED HOMEOBOX GENE 11* (*WOX11*) gene from a hybrid walnut (*JrWOX11*), which was found induced by abscisic acid (ABA), salt, and polyethylene glycol. Based on gene expression and overexpression studies, role of *JrWOX11* in improving salinity and osmotic tolerance through enhanced root system was demonstrated. This study provided the molecular basis of differentiating the trees that are difficult-to-root and easy-to-root. Li et al. characterized a novel NAC transcription factor (*NtNAC053*) in tobacco, which was induced by salt and drought stresses, localized in the nucleus, and acts as a transcriptional activator. This study demonstrated that enhanced tolerance to drought and salt stresses in *NtNAC053* overexpressing transgenic tobacco plants could be due to enhanced antioxidant system through activation of downstream stress-responsive genes. Lu et al. provided evidence for the existence of two isoforms, *ZmPP2C26L* and *ZmPP2C26S*, of *ZmPP2C26*, a clade B member of maize PP2C family. The *zmpp2c26* mutant exhibited enhanced drought tolerance, whereas overexpression of *ZmPP2C26L* and *ZmPP2C26S* significantly decreased drought tolerance in *Arabidopsis* and rice. The authors suggested dephosphorylation of ZmMAPK3 and ZmMAPK7 by the ZmPP2C26 protein as a possible mechanism to reduce drought tolerance and photosynthesis activity. The functional characterization of a valine-glutamine motif-containing gene in wheat, *TaVQ14*, was performed by Cheng et al. to reveal its role in salt and drought tolerance. The *TaVQ14* overexpressing lines in *Arabidopsis* showed improved salt and drought tolerance *via* scavenging reactive oxygen species and calcium signaling. Qin et al. focused on functional characterization of a transcription factor gene *HbMYB44* of rubber tree (*Hevea brasiliensis* Müll. Arg), which enhanced tolerance to salinity, drought, and osmotic stresses in overexpressing *Arabidopsis* plants. This gene also helped recovery of root damage in the overexpression plants by application of phytohormones such as ABA, methyl jasmonic acid, gibberellic acid, and salicylic acid which suggested *HbMYB44’*s versatile role in regulating multiple phytohormone signaling and stress tolerance pathways. Wang et al. demonstrated the role of Mitogen-activated Protein Kinase 1 in improving shading tolerance in rapeseed (*Brassica napus*) *via* increased photosynthetic capacity in *BnaMAPK1*-overexpressing plants. RNA sequencing revealed that *BnaMAPK1* positively regulated photosynthesis capability possibly by controlling antenna protein complex in photosystem II to respond to shading stress. Further, *BnaLHCB3* was identified as an interacting partner of BnaMAPK1 *via* yeast two-hybrid and split-luciferase complementation assays.

Jin et al. demonstrated that the ectopic expression of a soybean NHX gene, *GmNHX6* (encoding a Golgi-localized sodium/hydrogen exchanger), enhanced alkaline tolerance in *Arabidopsis* and soybean by maintaining high K^+^ content and low Na^+^/K^+^ ratio. A natural sequence variation in the promoter region of *GmNHX6* was associated with the alkaline tolerance in soybean germplasm and the promoter of *GmNHX6* isolated from an alkaline tolerant soybean variety exhibited stronger activity in response to alkali stress. In another study, Sun et al. utilized a T-DNA insertion mutant of *OsCIPK18* (*cipk18*) encoding a CBL-interacting protein kinase and defined an *OsCIPK18*-dependent transcriptomic network involved in ammonium toxicity response. In addition, the role of *OsCIPK18* as a key node in auxin and ABA signaling pathways under ammonium stress was proposed. Zhang et al. showed improvement in tolerance to drought and ABA in transgenic *Arabidopsis* plants overexpressing a glutathione S-transferase (*CsGSTU8*) from tea plant (*Camellia sinensis*). Further molecular insights were provided by demonstrating the binding of a trancription factor, CsWRKY48, to the promoter of *CsGSTU8* to regulate its induction under drought stress and ABA treatment.

## Genome-wide studies for discovery of candidate genes involved in cold/freezing stress

Cold and/or freezing stress is one of the major environmental factors, that limits the productivity of several plants. In the study by Wang et al., transcriptome profiling of leaves of two alfalfa genotypes with contrasting responses under freezing stress (-10°C) followed by co-expression network analysis revealed the importance of ATP-binding cassette (ABC) C subfamily genes, *ABCC8* and *ABCC3*, in freezing tolerance. Further, this study also demonstrated the contribution of Ca^2+^ signal transduction and CBF/DREB1 related genes towards tolerance to freezing stress. In another study, Islam et al. compared the transcriptional landscape in pseudostem and leaf blade tissues of endophyte-positive (E+) and endophyte-free (E−) tall fescue (*Festuca arundinacea*), a cool-season perennial grass, at three diurnal temperature conditions. The differential gene expression profiling revealed eight candidate genes, including orthologs of rice phytochrome A, phytochrome C, and ethylene receptor genes, which might be the possible route underlying freezing tolerance in tall fescue. A comparative transcriptomic analysis involving maize genotypes with contrasting response to low-temperature stress by Meng et al. revealed that both photosynthesis and antioxidant metabolism pathways played important role in conferring cold tolerance during seed germination stage. This was supported by data on increased antioxidant capacity in resistant line compared with the susceptible line. Further, Tang et al. demonstrated that cold treatment induced global DNA demethylation in *Hevea brasiliensis* and demethylation in the upstream regions of the genes was associated with higher gene expression.

## Studies addressing heavy metal stress response/tolerance

Heavy metal contamination not only reduces crop yield significantly, but also poses risks to human health. Identifying the molecular mechanisms heavy metal uptake can help in developing plants for phytoremediation as well as crops with reduced accumulation of such toxic metals. To investigate the molecular mechanism of cadmium (Cd) stress tolerance, Wang et al. performed RNA-seq analysis in *Tamarix hispida* treated with Cd stress for different time points. The functional annotation of differentially expressed genes identified genes involved in ion binding, signal transduction, stress sensing, hormone responses and ROS metabolism contributing toward Cd stress tolerance. Further, *ThUGT* from the ABA-signaling pathway was identified as a candidate gene to improve Cd stress tolerance by reducing Cd uptake and regulation of ROS. Another study by Paape et al. used GWAS approach with the seedlings of *Medicago truncatula* HapMap collection exposed to Cd and mercury (Hg) stress revealed significant genetic diversity for these phenotypic traits. Some important candidate genes in the QTL regions included, membrane associated ATP-binding cassette transporters, P-type ATPase transporters, oxidative stress response genes, and stress related UDP-glycosyltransferases, which can be the useful targets to design plants with reduced heavy metal accumulation. The study also suggested to exploit wild accessions of *Medicago* for genetic improvement due to macroevolutionary conservation of heavy metal and stress response genes in this model plant.

Lee et al. analyzed the interactions between arsenic (As) and eight essential ions in a rice core collection under non-stress and stress conditions to elucidate the impact of environmental and genotypic differences, and identified the genetic factors regulating As accumulation. This GWAS study provides evidence that *indica* populations are superior in reducing As accumulation compared with *japonica* populations. A potential candidate gene, *AIR2* (arsenic-induced RING finger protein), whose expression was lower in *indica* compared with *japonica* subspecies, was suggested for marker-assisted selection in developing rice varieties with improved grain quality.

## Studies providing insights into salinity and/or osmotic stress response/tolerance

A comparative analysis of transcriptomes of two contrasting clones (R7, salt-tolerant and S4, salt-sensitive) of *Fraxinus velutina* reported by Ma et al. revealed the upregulation of several stress-responsive genes in the salt-tolerant clone. Salt stress induced the expression of genes involved in proline biosynthesis, starch and sucrose metabolism, and those encoding antioxidant enzymes, which might contribute towards enhanced salt tolerance. Further, leaves and roots of both of these clones were subjected to miRNA and degradome analysis by Liu et al. to understand the role of miRNAs in defense response of plants to salt stress. This study revealed multiple and somewhat distinct miRNA/target modules regulating different biological processes in leaves (antioxidant system and auxin signaling) and roots (ROS scavenging, cell proliferation, and ion homeostasis) under salt stress. Based on GWAS of various growth and agronomic traits, genetic basis of salt-alkali tolerance was investigated by Zhang et al. and at least nine significant QTLs and 20 candidate genes related to salt-alkali stress tolerance were identified. By coupling the sequence variation, annotation and differential expression, few important candidate genes, such as *BnABA4, BnBBX14, BnVTI12, BnPYL8*, and *BnCRR1* were identified for breeding salt-alkali-tolerant *B. napus* varieties. Guan et al. identified five haplotypes of *GmSALT3* by using genome resequencing data from 279 Chinese soybean landraces. Using five PCR-based haplotype-specific markers developed in this study, the authors demonstrated their efficiency in distinguishing salt-tolerant and salt-sensitive soybean lines and tracing the salt-tolerant haplotype in soybean pedigree.

In a study by Galić et al., a link between biochemistry and genetics of osmotic stress tolerance in maize plants was established *via* investigating the variability in responses of a panel of elite maize inbred lines for the stress-related traits at the seedling stage. The overall analysis revealed genomic regions linked to stress responsive traits that harbor the genes associated with osmotic-stress signaling, osmolyte accumulation and regulation of peroxisomes gene ontology terms. The integrated transcriptomics and antioxidant profiling of two contrasting Chinese chestnut (*Castanea mollissima* BL.) varieties in response to gall wasp *Dryocosmus kuriphilus* (GWDK) infestation at different time points by Zhu et al. revealed new insights into the chestnut-GWKD interactions and identified candidate genes for further functional validation and molecular-aided breeding of gall wasp-resistant chestnut varieties.

The review article by Zhang et al. included in this Research Topic summarized the mechanisms associated with adaptative response to salinity, drought, and cold stresses as well as crosstalk among them in the model legume, *M. truncatula*. The genetic and molecular resources provided in this review should be useful for investigating and improving abiotic stress tolerance in legume crops. Future investigation on the impact of combination of abiotic stresses and use of wild species (*M. ruthenica*) is suggested for the retention of abiotic stress tolerance.

Due to increased awareness for improving sustainability of farming systems with minimal carbon footprints, it has become imperative to design climate smart crop varieties. However, accomplishing this goal is challenging due to involvement of multiple genes and pathways, and interactions among them. Despite tremendous advances made during last few decades, there are still gaps in comprehensive understanding of the plant’s response to abiotic stresses at molecular level. The collection of articles highlighting the relevance of genome-wide discovery of key genes associated with abiotic stress adaptation and their characterization in a wide range of plants using genomic approaches clearly contribute toward that goal. Since a tremendous number of natural variations for tolerance to abiotic stresses exists in the available cultivated and wild germplasm resources, future research should focus on mining of key abiotic stress related genes and their superior alleles, and gene pyramiding to develop crop varieties adapted to multiple stresses. The articles presented in this special issue not only enrich our understanding of the molecular basis of plants’ adaptative responses to abiotic stresses, but also should help in successful breeding of crop varieties adapted to climate-change using marker-assisted selection and genome editing tools.

## Author contributions

All authors listed have made a substantial, direct, and intellectual contribution to the work, and approved it for publication.
